# Breast cancer and NSAID use: a meta-analysis

**DOI:** 10.1054/bjoc.2000.1709

**Published:** 2001-05

**Authors:** S A Khuder, A B Mutgi

**Affiliations:** Department of Medicine, Medical College of Ohio, 3120 Glendale Ave, Toledo, Ohio 43614-5809, USA

**Keywords:** NSAID, breast cancer, meta-analysis, females

## Abstract

Recent epidemiological studies suggest that non-steroidal anti-inflammatory drugs (NSAIDs) reduce the risk of several cancers including breast cancer. This meta-analysis examined the studies on NSAID use and breast cancer. The estimators of relative risk and associated variances, which have been adjusted for the greatest number of confounders, were abstracted and included in the meta-analysis. Combined estimators of relative risk (RR) were calculated using either fixed or random effect models. Meta-analyses were performed on 6 cohort studies (number of cases ranged from 14 to 2414) and 8 case–control studies (number of cases ranged from 252 to 5882). The combined estimate of relative risk was 0.82 (95% confidence interval [CI] = 0.75–0.89). The combined estimate for cohort studies was 0.78 (95% CI = 0.62–0.99) and was 0.87 (95% CI = 0.84–0.91) for case–control studies. The findings of this meta-analysis suggest that NSAID use may be associated with a small decrease in the risk of breast cancer. However, the available data are insufficient to estimate the dose–response effect for duration and frequency of use of any particular types of NSAID. © 2001 Cancer Research Campaign http://www.bjcancer.com


*British Journal of Cancer* (2001) **84**(9), 1188–1192
© 2001 Cancer Research Campaign
doi: 10.1054/ bjoc.2001.1709, available online at http://www.idealibrary.com on IDEAL®

http://www.bjcancer.com

### Breast cancer and NSAID use: a meta-analysis

**SA Khuder and AB Mutgi**

Department of Medicine, Medical College of Ohio, 3120 Glendale Ave, Toledo, Ohio 43614-5809, USA

**Summary** Recent epidemiological studies suggest that non-steroidal anti-inflammatory drugs (NSAIDs) reduce the risk of several cancers including breast cancer. This meta-analysis examined the studies on NSAID use and breast cancer. The estimators of relative risk and associated variances, which have been adjusted for the greatest number of confounders, were abstracted and included in the meta-analysis. Combined estimators of relative risk (RR) were calculated using either fixed or random effect models. Meta-analyses were performed on 6 cohort studies (number of cases ranged from 14 to 2414) and 8 case–control studies (number of cases ranged from 252 to 5882). The combined estimate of relative risk was 0.82 (95% confidence interval [CI] = 0.75–0.89). The combined estimate for cohort studies was 0.78 (95% CI = 0.62–0.99) and was 0.87 (95% CI = 0.84–0.91) for case–control studies. The findings of this meta-analysis suggest that NSAID use may be associated with a small decrease in the risk of breast cancer. However, the available data are insufficient to estimate the dose–response effect for duration and frequency of use of any particular types of NSAID. © 2001 Cancer Research Campaign http://www.bjcancer.com

**Keywords**: NSAID; breast cancer; meta-analysis, females

Breast cancer is one of the most common cancers in women. The incidence of the disease has increased in recent years in the United States (Dinse et al, 1999). A substantial portion of the recent trend may be attributable to screening (Wun et al, 1995), however, the long-term trend remains unexplained. The established risk factors for breast cancer account for less than half of all breast cancer cases and offer few opportunities for intervention (Kelsey et al, 1993; Madigan et al, 1995).

Recently, non-steroidal anti-inflammatory drugs (NSAIDs) have received increasing attention due to their potential as chemopreventive agents against cancer. Animal studies showed inhibitory effect for NSAID on breast carcinogenesis (Lala et al, 1997; Robertson et al, 1998). However, several epidemiologic studies have examined the relation between NSAIDs and breast cancer, with inconsistent results. In some studies (Laakso et al, 1988; Gridley et al, 1993) patients with rheumatoid arthritis who use NSAIDs in high doses for symptom relief had fewer than expected cases of breast cancer.

Numerous studies have demonstrated that the level of prostaglandin is greater in breast cancer than in normal tissue (Bennett et al, 1983). In particular, the inducible form of cyclooxygenase (COX), the rate-limiting enzyme in prostaglandin biosynthesis may be overexpressed in breast cancer (Hwang et al, 1998). NSAIDs are known to block COX activity (Robertson et al, 1998) and thus become attractive agents for breast cancer prevention. A recent animal study (Harris et al, 2000) confirmed the chemopreventive activity of NSAIDs against breast cancer through COX2 blocking. In this meta-analysis we examined the epidemiological studies on NSAID use and breast cancer.

*Received 19 October 2000*
*Revised 11 January 2001*
*Accepted 16 January 2001*

*Correspondence to:* Sadik A. Khuder

### METHODS

The studies were located via a search of the MEDLINE (from 1966 to 2000) and Cancer Abstract databases (from 1980 to 2000). Abstracts of research presented at related conferences (Society for Epidemiologic Research, European Cancer Research, British Cancer Research, and American Association for Cancer Research) were also searched.

Studies were eliminated from the analyses if they included subjects used in other more-inclusive studies. The estimators of relative risk and associated variances, which has been adjusted for the greatest number of confounders, were abstracted and included in the meta-analysis.

A series of meta-analyses was conducted and the results were evaluated in the context of the published literature. The homogeneity of the estimators of relative risk was tested using Cochran's Q statistics (Cochran, 1954). This is a chi-square test with degrees of freedom equal to the number of studies minus one, and tests the null hypothesis that the within-study estimates of relative risk are homogeneous across studies. The fixed-effect model was used to obtain the combined estimator of relative risk and its standard error (SE). The random-effects model (DerSimonian and Laird, 1987) was used in situation when we detected significant heterogeneity within the groups of studies.

The potential for publication bias in published reports was investigated by constructing funnel plots of log odds ratio against the size of the study. A Kendall tau rank correlation test (Begg and Mazumdar, 1994) was used to test for the statistical significance of publication bias.

### RESULTS

We identified 15 studies (Friedman and Ury, 1980; Paganini-Hill et al, 1989; Rosenberg et al, 1991; Thun et al, 1993; Schreinemachers and Emerson, 1994; Harris et al, 1995; Rosenberg, 1995; Egan et al, 1996; Harris et al, 1996; Neugut et al, 1998; Coogan et al, 1999; Harris et al, 1999; Cotterchio et al, 2000;

**Table 1** Cohort studies used for meta-analyses of NSAIDs use and breast cancer

| Reference | NSAID Ttype | Number of cases | OR | 95% CI |
|---|---|---|---|---|
| Harris et al, 1999 | Aspirin | 76 | 0.60 | 0.47–0.77 |
| | Acetaminophen | 36 | 0.84 | 0.60–1.18 |
| | Ibuprofen | 37 | 0.51 | 0.36–0.72 |
| Egan et al, 1996 | Aspirin | 2414 | 1.01 | 0.80–1.27 |
| Schreinemachers and Everson, 1994 | Aspirin | 79 | 0.70 | 0.50–0.96 |
| Thun et al, 1993 | Aspirin | – | 0.94 | 0.80–1.10 |
| Paganini-Hill et al, 1989 | Aspirin | 68 | 0.96 | 0.75–1.21 |
| Friedman and Ury, 1980 | Aspirin, | 2 | 0.20 | 0.05–0.80 |
| | Indomethacin | 12 | 0.50 | 0.28–0.88 |

**Table 2** Case–control studies used for meta-analyses of NSAIDs use and breast cancer

| Reference | Number of cases | NSAID type | Type of control | OR | 95% CI |
|---|---|---|---|---|---|
| Sharpe et al, 2000[a] | 5882 | Any | General population | 0.90 | 0.84–0.97 |
| Cotterchio et al, 2000 | 2681 | Any | General population | 0.74 | 0.65–0.85 |
| Langman et al, 2000 | 3105 | Any | Cancer | 1.00 | 0.92–1.09 |
| Coogan et al, 1999 | 6558 | Any | Cancer | 0.80 | 0.70–1.00 |
| | | | Non-cancer | 0.70 | 0.60–0.90 |
| | | Aspirin | Cancer | 0.70 | 0.60–0.90 |
| | | | Non-cancer | 0.70 | 0.50–0.80 |
| Neugut et al, 1998 | 252 | Aspirin | Non-cancer | 0.80 | 0.35–1.80 |
| Harris et al, 1996 | 106 | Any | General population | 0.66 | 0.52–0.83 |
| | | Aspirin, | General population | 0.69 | 0.46–0.99 |
| | | Ibuprofen | General population | 0.57 | 0.36–0.91 |
| Roenberg, 1995 | 4485 | Any | Cancer | 0.90 | 0.60–1.20 |
| | | | Non-cancer | 0.80 | 0.60–1.00 |
| Harris et al, 1995 | 744 | Any | Cancer | 0.96 | 0.67–1.39 |
| | | | Non-cancer | 0.81 | 0.63–1.03 |

[a]Nested case–control study.

**Table 3** Combined analysis of studies used for meta-analysis of NSAID use and breast cancer

| Reference | NSAID type | Number of studies | Test of heterogeneity | | OR | 95% CI |
|---|---|---|---|---|---|---|
| | | | $\chi^2$ | *P* value | | |
| All studies | Any | 16 | 38.1 | 0.001 | 0.80 | 0.73–0.87 |
| Cohort studies | Any | 6 | 22.8 | <0.001 | 0.78 | 0.62–0.99 |
| | Aspirin | 6 | 17.8 | <0.001 | 0.79 | 0.59–1.06 |
| Case–control | | | | | | |
| All | Any | 10 | 15.2 | 0.09 | 0.83 | 0.79–0.88 |
| Cancer | | 3 | 1.0 | 0.621 | 0.84 | 0.72–0.97 |
| Non-cancer | | 7 | 14.2 | 0.03 | 0.79 | 0.72–0.86 |
| All | Aspirin | 4 | 0.10 | 0.99 | 0.70 | 0.61–0.81 |

Langman, 2000; Sharpe et al, 2000) evaluating the association between NSAIDs and breast cancer that were published between 1980 and 2000. One study (Rosenberg et al, 1991) was excluded from the analysis because of lack of data on the estimator of relative risk. The remaining studies were 6 cohort studies (Table 1) and 8 case–control studies (Table 2). The number of cases ranged from 14 to 2414 for cohort studies. For case–control studies the number of cases ranged from 252 to 5882 and the number of controls ranged from 42 to 89528. 5 studies (Paganini-Hill et al, 1989; Thun et al, 1993; Schreinemachers and Emerson, 1994; Egan et al, 1996; Neugut et al, 1998) were restricted to aspirin use and three studies (Harris et al, 1999; Paganini-Hill et al, 1989; Friedman and Ury, 1980) provided data stratified by NSAID type.

Twelve studies reported reduction in the risk of breast cancer with NSAID use. The estimator of relative risk for cohort studies ranged from 0.20 to 1.01 and 6 of these estimators were significant. Only one cohort study (Egan et al, 1996) reported non-significant increases in the risk of breast cancer with aspirin use. The estimator of relative risk for case–control studies ranged from 0.57 to 1.00 and 7 of these estimators were significant. None of the case–control studies reported an increase in the risk of breast cancer with any NSAID use.

The results of meta-analyses are presented in Table 3. Significant heterogeneity was detected among the studies ($\chi^2$ = 53.0, $P$ = 0.001). The combined estimate of relative risk using the random-effect model was 0.82 (95% CI = 0.75–0.89). Heterogeneity among studies was significantly reduced when studies were combined within specific design and type of control. The combined estimate of relative risk for cohort studies was 0.78 (95% CI 0.62–0.99) with any NSAIDs and was 0.79 (95%

*© 2001 Cancer Research Campaign* 

**Table 4** Duration of use reported in studies used in the meta-analysis of NSAID use and breast cancer

| Study | Measure | Duration | OR | 95% CI |
|---|---|---|---|---|
| Coogan et al, 1999[a] | NSAID regular use (years) begun ≥ 1 year before admission | Never | 1 | – |
| | | < 1 | 0.90 | 0.50–1.70 |
| | | 1–< 2 | 1.10 | 0.70–1.70 |
| | | 2–< 5 | 0.70 | 0.50–1.00 |
| | | 5–< 10 | 0.70 | 0.40–1.00 |
| | | 10–< 20 | 0.70 | 0.40–1.10 |
| | | 20+ | 0.60 | 0.30–1.00 |
| | | Unknown | 0.40 | 0.20–0.70 |
| Harris et al, 1995 | NSAID use, years | 0 | 1 | – |
| | | 1–4 | 1.09 | 0.80–1.50 |
| | | ≥5 | 0.63 | 0.50–0.90 |
| Egan et al, 1996 | Aspirin use, years | <5 | 0.89 | 0.76–1.05 |
| | | 5–9 | 0.98 | 0.81–1.19 |
| | | 10–19 | 1.11 | 0.85–1.46 |
| | | ≥20 | 1.00 | 0.71–1.41 |
| Harris et al, 1996 | NSAID use, years | <5 | 0.65 | 0.47–0.91 |
| | | ≥5 | 0.60 | 0.40–0.91 |
| Langman et al, 2000 | Prescription of NSAID before diagnosis, months | 13–24 | 1.03 | 0.93–1.13 |
| | | 25–36 | 1.00 | 0.91–1.11 |
| Sharpe et al, 2000 | Highest level of NSAID exposure before diagnosis, years | 1/2 | 1.05 | 0.91–1.23 |
| | | 1/2–1 | 1.20 | 1.02–1.40 |
| | | 2–5 | 0.76 | 0.63–0.92 |
| | | 6–10 | 1.13 | 0.92–1.39 |
| | | 11–15 | 0.83 | 0.63–1.11 |

[a]Significant dose–response relationship.

**Table 5** Frequency of NSAID use in studies used in the meta-analysis of NSAID use and breast cancer

| Study | Measure | Frequency | OR | 95% CI |
|---|---|---|---|---|
| Sharpe et al, 2000[a] | Sum of NSAID mg day$^{-1}$ dispensed ÷ maximum mg day$^{-1}$ 0 recommended (Σp) in 2–5 years before diagnosis | 1.0 | – | |
| | | 0 < Σp ≤ 1 | 0.93 | 0.85–1.01 |
| | | 0.1 < Σp ≤ 0.3 | 0.91 | 0.79–1.06 |
| | | Σp >0.3 | 0.76 | 0.63–0.92 |
| Egan et al, 1996 | Number of aspirins per week | 0 | 1.00 | – |
| | | 1–3 | 0.99 | 0.89–1.11 |
| | | 4–6 | 0.94 | 0.80–1.10 |
| | | 7–10 | 1.00 | 0.84–1.20 |
| | | 11–14 | 1.11 | 0.91–1.37 |
| | | >14 | 1.05 | 0.89–1.23 |
| Paganini-Hill et al, 1989 | Aspirin use | None | 1 | – |
| | | <daily | 0.95 | 0.68–1.34 |
| | | daily | 0.96 | 0.69–1.34 |
| Harris et al, 1996 | NSAID dose/week | 3–6 | 0.73 | 0.46–1.13 |
| | | ≥7 | 0.63 | 0.49–0.81 |
| Thun et al, 1993 | Aspirin frequency/month | occasionally | 0.93 | 0.73–1.19 |
| | | 1–15 | 0.98 | 0.76–1.26 |
| | | 16+ | 0.88 | 0.62–1.24 |
| Harris et al, 1999 | NSAID pills per week | 0 < 1 | 1.00 | – |
| | | 1–3 | 0.64 | 0.50–0.82 |
| | | ≥4 | 0.57 | 0.44–0.74 |
| Langman et al, 2000 | Number of prescriptions of NSAID received in 13–24 months before diagnosis | 0 | 1 | – |
| | | 1 | 0.99 | 0.87–1.13 |
| | | 2–6 | 0.96 | 0.83–1.11 |
| | | ≥7 | 1.10 | 0.92–1.30 |

[a]Significant dose–response relationship.

CI = 0.59–1.06) for aspirin use. The case–control studies were relatively homogeneous within cancer and non-cancer controls. The combined estimate of relative risk for these studies was 0.87 (95% CI 0.84–0.91) with any NSAID and was 0.70 (95% CI = 0.61–0.81) for aspirin use. The combined estimate of relative risk for studies with non-cancer controls was 0.79 (95% CI 0.72–0.86) and was 0.96 (95% CI = 0.89–1.03) for studies with cancer controls.

 *© 2001 Cancer Research Campaign*

Six studies provided results on duration of use of aspirin and that of other NSAIDs (Table 4). 9 studies provided results stratified by measures of NSAID use (Table 4). The risk reduction for highest duration of use (20+ years) ranged from 40% (Coogan et al, 1999) to zero (Egan et al, 1996). Trend tests for dose–response relations in only one of these studies was significant. The available data in Table 4 are insufficient to estimate the combined dose–response effect for duration of use of any particular types of NSAID.

Seven studies provided results on frequency of use of aspirin and that of other NSAID (Table 5). In one study (Harris et al, 1999) four or more pills of NSAID per week was associated with a 43% reduction in the risk of breast cancer (RR = 0.57, 95% CI 0.44–0.74). In another study (Paganini-Hill et al, 1989) daily use of aspirin was associated with only 4% reduction in the estimated risk of breast cancer (RR = 0.96, 95% CI 0.69–1.34). The available data in Table 5 are insufficient to estimate the combined dose–response effect for frequency of use of any particular types of NSAID.

There was no evidence of publication bias in studies included in this meta-analysis. The Kendall tau correlation coefficient for the standard error and the standardized log odds ratio was 0.96 ($P = 0.34$).

### DISCUSSION

This meta-analysis showed that NSAID use may decrease the risk of breast cancer. This is evident by the consistently reduced relative risk in the majority of studies included in the analysis. The effect observed was similar in most studies regardless of design or type of cases (incident or fatal cases). The only negative study (Egan et al, 1996) may have been confounded by reproductive factors. In this meta-analysis, regular use of NSAIDs was associated with an 18% reduction in the risk of breast cancer. The reduction in risk was higher in cohort studies (21%) than case–control studies (13%). Within case–control studies, the reduction in risk was smaller in studies with cancer controls than in those with non-cancer controls. Although this finding is consistent with studies on NSAID use and colon cancer (Harris et al, 1995), it may argue against a true effect against breast cancer since this should be consistent across control groups. It is possible that some cancer subtypes (for example, gastrointestinal) were related to NSAID use and these patients discontinued the drug. If so this may overestimate the odds ratio and may bias the estimate of relative risk away from the null value. It is possible that the results of these studies are biased by a higher prevalence of pre-existing medical conditions commonly associated with NSAID use among non-cancer controls. On the other hand population-based case–control studies (Neugut et al, 1998) used as control subjects who underwent screening mammography, and their use of NSAID could have overestimated the prevalence of use in the study base.

With regard to type of NSAID, aspirin was the major type used in the studies included in this meta-analysis. In general, the reduction in risk of breast cancer with aspirin use was similar to other NSAID type. Two studies reported higher reduction in risk with ibuprofen in comparison to aspirin. However, available data were not adequate enough to test this in a meta-analysis.

Nine studies evaluated dose–response relation of NSAID use and breast cancer but only two studies reported significant dose–response relation for duration (Coogan et al, 1999) and frequency (Sharpe et al, 2000) of NSAID use. In one study (Coogan et al, 1999) the highest reduction in breast cancer risk was reported for the category ‘unknown years of use’.

Although publication bias is possible because of the possibility of failure of investigators to submit negative results or failure of journals to publish negative studies our analysis did not suggest this.

The effect of NSAIDs on breast cancer risk reduction is biologically plausible. A number of animal studies have suggested a protective effect of NSAIDs against mammary cancer. A potential mechanism for anti-tumour effect of NSAIDs involves inhibition of the synthesis of prostaglandins. NSAIDs block the enzyme cyclooxygenase and in turn inhibit prostaglandin biosynthesis. Prostaglandins may serve as cofactors in carcinogenesis with potential effects ranging from direct mutagenesis to tumour promotion and immune suppression (Lupulescu, 1978; Mellemkjaer et al, 1996). One study (McCormick and Wilson, 1986) suggests that the cancer inhibitory effects of NSAIDs may be independent of their effects on prostaglandin synthesis. There is evidence from animal studies that indomethacin inhibits the effects of oestrogen in the pituitary gland (Neugut et al, 1998). In-vitro studies of human breast cancer cells indicate that acetylsalicylic acid may inhibit direct binding of oestradiol to oestrogen receptor (Thompson et al, 1995).

The majority of studies included in this meta-analysis adjusted for known risk factor for breast cancer. Our inclusion of estimators of relative risk, which were adjusted for the greatest number of confounders, would have reduced the possibility of confounding effect. The combined estimate of this study supports a protective effect for NSAIDs against breast cancer. Other support for the protective effect of NSAIDs against breast cancer comes from studies on patients with rheumatoid arthritis who use NSAIDs in high doses for symptom relief. 2 studies (Gridley et al, 1993; Baron, 1995) reported that these patients had less than expected occurrence of breast cancer. The risk pattern for NSAID users found in this meta-analysis is consistent with the pattern found in studies on patients with rheumatoid arthritis.

The limitations of this study stem from the studies included in the meta-analysis. All the studies included are observational studies and therefore subject to biases. Some are case–control studies and we cannot rule out the possibility of selection and information biases. It is possible that the relation between NSAIDs and breast cancer may reflect a recall bias by the cases or controls. Misclassification of exposure is a potential problem in observational epidemiological studies. None of the studies included in this meta-analysis utilized an objective method of exposure assessment. In all studies the NSAID use was self-reported and therefore subject to recall bias. These drugs are often taken sporadically in a pattern of intake that may be difficult to remember or summarize for some subjects. For example, some widely used brands or combination product may not be recognized as containing aspirin (Harris et al, 1995). It is possible that NSAID use may reflect a health consciousness among the control group. However, Harris et al (1995) reported no association between NSAID use and level of education, which can be taken as a proxy for health consciousness.

Currently known risk factors account for less than half of all breast cancer cases and offer limited opportunities for intervention. Therefore, any preventive measure identified will be important. This meta-analysis suggests that NSAIDs have a weak chemopreventive value against breast cancer. However, we cannot rule out the possibility of an alternate explanation for this finding due to the limitations of the studies included in the analysis.

There is a need for more studies that prospectively evaluate the reduction in risk of breast cancer utilizing a better measure of NSAID dose. There is a need to establish whether NSAIDs are

© 2001 Cancer Research Campaign efficacious in preventing breast cancer and type and optimal dose. This can be accomplished using a randomized clinical trial on different types of NSAID.

### REFERENCES

Baron JA (1995) Aspirin and cancer. *Prev Med* **24**: 121–124

Bennett A, Berstock DA, Carroll MA, Stamford IF and Wilson AJ (1983) Breast cancer, its recurrence, and patient survival in relation to tumor prostaglandins. In: Samuelsson B, Paoletti R, Ramwell P (eds) *Advances in prostaglandin, thromboxane, and leukotriene research*, Vol 12. Raven Press: New York

Begg CB and Mazumdar M (1994) Operating characteristics of a rank correlation test for publication bias. *Biometrics* **50**: 1088–1101

Carter CA, Milholland RJ, Shea W and Ip MM (1983) Effect the prostaglandin synthetase inhibitor indomethacin on 7, 12-dimethylbenz(a)anthracene-induced mammary tumorigenesis in rats fed different levels of fat. *Cancer Res* **43**: 3559–3562

Cochran WG (1954) The combination of estimates from different experiments. *Biometrics* **8**: 101–129

Coogan FP, Rao RS, Rosenberg L, Palmer JR, Strom BL, Zauber AG, Stolley PD and Shapiro S (1999) The relationship of nonsteroidal anti-inflammatory drug use to the risk of breast cancer. *Prev Med* **29**: 72–76

Cotterchio M, Kreiger N, Steingart A and Buchan G (2000) Non-steroidal anti-inflammatory drugs (NSAID) use and breast cancer. SER Abstract. *Am J Epidemiol* **151**: S72

DerSimonian R and Laird N (1987) Meta-analysis in clinical trials. *Controlled Clin Trials* **7**: 177–188

Dinse GE, Umbach DM, Sasco AJ, Hoel DG and Davis DL (1999) Unexplained increases in cancer incidence in the United States from 1975 to 1994: possible sentinel health indicators? *Ann Rev Public Health* **20**: 173–209

Egan KM, Stampfer MJ, Giovannucci E, Rosner BA and Colditz GA (1996) Prospective study of regular aspirin use and the risk of breast cancer. *J Natl Cancer Inst* **88**: 988–993

Friedman GD and Ury HK (1980) Initial screening for carcinogenicity of commonly used drugs. *J Natl Cancer Inst* **65**: 723–733

Gridley G, McLaughlin JK, Ekbom A, Klareskog L, Adami H, Hacker DG, Hoover R and Fraumeni Jr JF (1993) Incidence of cancer among patients with rheumatoid arthritis. *J Natl Cancer Inst* **85**: 307–311

Harris RE, Namboodiri K, Stellman SD and Wynder EL (1995) Breast cancer and NSAID use: heterogeneity of effect in a case-control study. *Prev Med* **24**: 119–120

Harris RE, Namboodiri KK and Farrar WB (1996) Nonsteroidal antiinflammatory drugs and breast cancer. *Epidemiology* **7**: 203–205

Harris RE, Kasbari S and Farrar WB (1999) Prospective study of nonsteroidal anti-inflammatory drugs and breast cancer. *Oncol Rep* **6**: 71–73

Harris RE, Alshafie GA and Seibert K (2000) Chemoprevention of breast cancer in rats by celecoxib, a cyclooxygenase 2 inhibitor. *Cancer Res* **60**: 2101–2103

Hwang D, Scollard D, Byrne J and Levine E (1998) Expression of cyclooxygenase-1 and cyclooxygenase-2 in human breast cancer. *J Natl Cancer Inst* **90**: 455–460

Kelsey JL, Gammon MD and John EM (1993) Reproductive factors and breast cancer. *Epidemiol Rev* **15**: 36–47

Laakso M, Mutru O, Isomaki H and Koota K (1988) Cancer mortality in patients with rheumatoid arthritis. *J Rheumatol* **15**: 1319–1322

Lala PK, Al-mutter N and Orucevic A (1997) Effects of chronic indomethacin therapy on the development and progressions of spontaneous mammary tumors in C3H/HEJ mice. *Int J Cancer* **73**: 371–380

Langman MJS, Cheng KK, Gilman EA, Lancashire RJ (2000) Effect of anti-inflammatory drugs on the overall risk of common cancer: case-control study in general practice research database. *Br Med J* **320**: 1642–1646

Lupulescu A (1978) Enhancement of carcinogenesis by prostaglandins. *Nature* **272**: 634–636

Lynch NR, Castes M, Astoin M and Salomon JC (1978) Mechanism of inhibition of tumour growth by aspirin and indomethacin. *Br J Cancer* **38**: 503–512

Madigan MP, Ziegler RG, Benichou J, Byrne C and Hoover RN (1995) Proportion of breast cancer cases in the United States explained by well-established risk factors. *J Natl Cancer Inst* **87**: 1681–1685

McCormick DI and Wilson AM (1986) Combination chemoprevention of rat mammary carcinogenesis by indomethacin. *Cancer Res* **46**: 3907–3911

Mellemkjaer L, Linet MS, Gridley G, Frisch M, Moller H and Olsen JH (1996) Rheumatoid arthritis and cancer risk. *Eur J Cancer* **32A**: 1753–1757

Neugut AI, Rosenberg DJ, Ahsan H, Jacobson JS, Wahid N, Hagan M, Rahman MI, Khan ZR, Chen L, Pablos-Mendez A and Shea S (1998) Association between coronary heart disease and cancers of the breast, prostate, and colon. *Cancer Epidemiol Biomarker Prev* **7**: 869–873

Paganini-Hill A, Chao A, Ross RK and Henderson BE (1989) Aspirin use and chronic diseases: a cohort study of the elderly. *Br Med J* **299**: 1247–1250

Robertson FM, Parett ML, Jorder FS, Ross M, Abo-Issa, Alshafie G and Harris RE (1998) Ibuprofen-induced inhibition of cyclooxygenase isoform gene expression and regression of rat mammary carcinoma. *Cancer Lett* **122**: 165–175

Rosenberg L (1995) Nonsteroidal anti-inflammatory drugs and cancer. *Prev Med* **24**: 107–109

Rosenberg L, Palmer JR, Zauber AG, Warshauer ME, Stolley PD and Shapiro S (1991) A hypothesis: nonsteroidal anti-inflammatory drugs reduce the incidence of large-bowel cancer. *J Natl Cancer Inst* **83**: 355–358

Schreinemachers DM and Everson RB (1994) Aspirin use and lung, colon, and breast cancer incidence in a prospective study. *Epidemiology* **5**: 138–146

Sharpe CR, Collet JP, McNutt M, Belzille E, Boivin JF and Hanley JA (2000) Nested case-control study of the effects of non-steroidal anti-inflammatory drugs on breast cancer risk and stage. *Br J Cancer* **83**: 112–120

Thompson HJ, Briggs S, Paranka NS, Piazza GA, Brendel K, Gross PH, Sperl GJ, Pamukcu R and Ahnen DJ (1995) Inhibition of mammary carcinogenesis in rats by sulfone metabolite of sulindac *J Natl Cancer Inst* **87**: 1259–1260

Thun MJ, Namboodri MM, Calle EE, Flanders WD and Heath CW (1993). Aspirin use and risk of fetal cancer. *Cancer Res* **53**: 1322–1327

Van Aswegen C, van Schalkwyk ICD, Roux LJ, Becker PJ and Du Plessis DJ (1992) A novel study on the effect of acetylsalicylic acid on the binding capacity of estrogen receptors from MCF-7 cells. *Clin Physiol Biochem* **9**: 145–149

Wun LM, Feuer EJ and Miller BA (1995) Are increases in mammographic screening still a valid explanation for trend in breast cancer incidence in the United States? *Cancer Cause Control* **6**: 135–144

*© 2001 Cancer Research Campaign*

## References

[PDF_00362] Baron J. A. (1995). Aspirin and cancer.. Prev Med.

[PDF_00367] Begg C. B., Mazumdar M. (1994). Operating characteristics of a rank correlation test for publication bias.. Biometrics.

[PDF_00369] Carter C. A., Milholland R. J., Shea W., Ip M. M. (1983). Effect of the prostaglandin synthetase inhibitor indomethacin on 7,12-dimethylbenz(a)anthracene-induced mammary tumorigenesis in rats fed different levels of fat.. Cancer Res.

[PDF_00375] Coogan P. F., Rao S. R., Rosenberg L., Palmer J. R., Strom B. L., Zauber A. G., Stolley P. D., Shapiro S. (1999). The relationship of nonsteroidal anti-inflammatory drug use to the risk of breast cancer.. Prev Med.

[PDF_00381] DerSimonian R., Laird N. (1986). Meta-analysis in clinical trials.. Control Clin Trials.

[PDF_00383] Dinse G. E., Umbach D. M., Sasco A. J., Hoel D. G., Davis D. L. (1999). Unexplained increases in cancer incidence in the United States from 1975 to 1994: possible sentinel health indicators?. Annu Rev Public Health.

[PDF_00386] Egan K. M., Stampfer M. J., Giovannucci E., Rosner B. A., Colditz G. A. (1996). Prospective study of regular aspirin use and the risk of breast cancer.. J Natl Cancer Inst.

[PDF_00389] Friedman G. D., Ury H. K. (1980). Initial screening for carcinogenicity of commonly used drugs.. J Natl Cancer Inst.

[PDF_00391] Gridley G., McLaughlin J. K., Ekbom A., Klareskog L., Adami H. O., Hacker D. G., Hoover R., Fraumeni J. F. (1993). Incidence of cancer among patients with rheumatoid arthritis.. J Natl Cancer Inst.

[PDF_00400] Harris R. E., Alshafie G. A., Abou-Issa H., Seibert K. (2000). Chemoprevention of breast cancer in rats by celecoxib, a cyclooxygenase 2 inhibitor.. Cancer Res.

[PDF_00398] Harris R. E., Kasbari S., Farrar W. B. (1999). Prospective study of nonsteroidal anti-inflammatory drugs and breast cancer.. Oncol Rep.

[PDF_00396] Harris R. E., Namboodiri K. K., Farrar W. B. (1996). Nonsteroidal antiinflammatory drugs and breast cancer.. Epidemiology.

[PDF_00394] Harris R. E., Namboodiri K., Stellman S. D., Wynder E. L. (1995). Breast cancer and NSAID use: heterogeneity of effect in a case-control study.. Prev Med.

[PDF_00402] Hwang D., Scollard D., Byrne J., Levine E. (1998). Expression of cyclooxygenase-1 and cyclooxygenase-2 in human breast cancer.. J Natl Cancer Inst.

[PDF_00404] Kelsey J. L., Gammon M. D., John E. M. (1993). Reproductive factors and breast cancer.. Epidemiol Rev.

[PDF_00408] Lala P. K., Al-Mutter N., Orucevic A. (1997). Effects of chronic indomethacin therapy on the development and progression of spontaneous mammary tumors in C3H/HEJ mice.. Int J Cancer.

[PDF_00411] Langman M. J., Cheng K. K., Gilman E. A., Lancashire R. J. (2000). Effect of anti-inflammatory drugs on overall risk of common cancer: case-control study in general practice research database.. BMJ.

[PDF_00414] Lupulescu A. (1978). Enhancement of carcinogenesis by prostaglandins.. Nature.

[PDF_00416] Lynch N. R., Castes M., Astoin M., Salomon J. C. (1978). Mechanism of inhibition of tumour growth by aspirin and indomethacin.. Br J Cancer.

[PDF_00418] Madigan M. P., Ziegler R. G., Benichou J., Byrne C., Hoover R. N. (1995). Proportion of breast cancer cases in the United States explained by well-established risk factors.. J Natl Cancer Inst.

[PDF_00421] McCormick D. L., Wilson A. M. (1986). Combination chemoprevention of rat mammary carcinogenesis by indomethacin and butylated hydroxytoluene.. Cancer Res.

[PDF_00423] Mellemkjaer L., Linet M. S., Gridley G., Frisch M., Møller H., Olsen J. H. (1996). Rheumatoid arthritis and cancer risk.. Eur J Cancer.

[PDF_00425] Neugut A. I., Rosenberg D. J., Ahsan H., Jacobson J. S., Wahid N., Hagan M., Rahman M. I., Khan Z. R., Chen L., Pablos-Mendez A. (1998). Association between coronary heart disease and cancers of the breast, prostate, and colon.. Cancer Epidemiol Biomarkers Prev.

[PDF_00429] Paganini-Hill A., Chao A., Ross R. K., Henderson B. E. (1989). Aspirin use and chronic diseases: a cohort study of the elderly.. BMJ.

[PDF_00431] Robertson F. M., Parrett M. L., Joarder F. S., Ross M., Abou-Issa H. M., Alshafie G., Harris R. E. (1998). Ibuprofen-induced inhibition of cyclooxygenase isoform gene expression and regression of rat mammary carcinomas.. Cancer Lett.

[PDF_00435] Rosenberg L. (1995). Nonsteroidal anti-inflammatory drugs and cancer.. Prev Med.

[PDF_00437] Rosenberg L., Palmer J. R., Zauber A. G., Warshauer M. E., Stolley P. D., Shapiro S. (1991). A hypothesis: nonsteroidal anti-inflammatory drugs reduce the incidence of large-bowel cancer.. J Natl Cancer Inst.

[PDF_00440] Schreinemachers D. M., Everson R. B. (1994). Aspirin use and lung, colon, and breast cancer incidence in a prospective study.. Epidemiology.

[PDF_00443] Sharpe C. R., Collet J. P., McNutt M., Belzile E., Boivin J. F., Hanley J. A. (2000). Nested case-control study of the effects of non-steroidal anti-inflammatory drugs on breast cancer risk and stage.. Br J Cancer.

[PDF_00406] Symmons D. P. (1988). Neoplasia in rheumatoid arthritis.. J Rheumatol.

[PDF_00446] Thompson H. J., Briggs S., Paranka N. S., Piazza G. A., Brendel K., Gross P. H., Sperl G. J., Pamukcu R., Ahnen D. J. (1995). Inhibition of mammary carcinogenesis in rats by sulfone metabolite of sulindac.. J Natl Cancer Inst.

[PDF_00449] Thun M. J., Namboodiri M. M., Calle E. E., Flanders W. D., Heath C. W. (1993). Aspirin use and risk of fatal cancer.. Cancer Res.

[PDF_00455] Wun L. M., Feuer E. J., Miller B. A. (1995). Are increases in mammographic screening still a valid explanation for trends in breast cancer incidence in the United States?. Cancer Causes Control.

[PDF_00451] van Aswegen C., Dirksen van Schalkwyk J. C., Roux L. J., Becker P. J., Du Plessis D. J. (1992). A novel study on the effect of acetylsalicylic acid on the binding capacity of estrogen receptors from MCF-7 cells.. Clin Physiol Biochem.

